# Calcifying Epithelial Odontogenic (Pindborg) Tumor in a Child: A Case Report and Literature Review

**DOI:** 10.1007/s12105-019-01009-1

**Published:** 2019-02-15

**Authors:** Shoreh R. Fazeli, Kamyar R. Giglou, Mahmoud L. Soliman, Waleed H. Ezzat, Andrew Salama, Qing Zhao

**Affiliations:** 1 Department of Pathology, Boston University, Boston Medical Center, Boston, MA USA; 2 Department of Oral and Maxillofacial Surgery, Boston University, School of Dental Medicine, Boston, MA USA; 3 Department of Otolaryngology-Head and Neck, Boston University, Boston Medical Center, Boston, MA USA

## Abstract

Calcifying epithelial odontogenic tumor (CEOT) is a rare neoplasm, which accounts for < 1% of all odontogenic tumors. CEOT occurs more frequently in adults with a peak incidence in the 5th decade of life and is extremely rare in the pediatric population. We present a case of a 13-year-old girl who was found to have a mandibular CEOT. We summarize the radiological features, pathological findings, clinical management and literature review focusing on this entity in children.

**Electronic supplementary material:**

The online version of this article (10.1007/s12105-019-01009-1) contains supplementary material, which is available to authorized users.

## Introduction

The calcifying epithelial odontogenic tumor (CEOT), also known as Pindborg tumor, is a rare and typically benign odontogenic neoplasm [1]. Danish pathologist Jens J. Pindborg first described it as a separate entity in 1958. He reported 3 cases; all male patients with the age ranging from 40 to 53 years [2]. Two of the three patients had recurrent tumors, of which one recurred 2 months and one 6 years after the initial excisions. CEOT most commonly occurs in individuals between 20 and 60 years of age, with peak incidence in the 5th decade; however a wide age range from 8 to 92 years has been reported [1, 3]. To date, about 200 cases have been reported [4], of which only 14 cases including the present case occurred in children [1, 5–15]. Although this tumor does not show a gender predilection, 71% of the cases reported in children have been seen in females (Table 1). The most common location of the tumor is the mandibular premolar and molar region (68%) and, less frequently, the maxilla [16–19]. Half of the cases are associated with an impacted tooth [17, 20, 21]. Clinically, CEOT can be found incidentally or may present as a slowly growing mass. Radiologically, the lesion appears radiolucent with variable calcification and can have unilocular or multilocular cystic appearance. These findings are not specific and simulate an ameloblastoma, dentigerous cyst, or other odontogenic tumors. Although typically benign, CEOT tends to invade local structures and has a potential for recurrence. Nevertheless, malignant CEOT or malignant transformation and distant metastasis have been reported only in adults but are extremely rare [22–24]. Surgical resection with negative margins to minimize the risk of recurrence and long-term follow-up is the management of choice. Herein we present a case of a large CEOT in a 13-year-old girl, together with a literature review focusing on the pediatric group.

**Table 1:** Reported cases of CEOT in children

Author	Age (year); gender	Size(cm); location	Amyloid; Congo red	Impacted/or developing tooth	Follow-up period
Akhtar et al. [1]	16; M	6.5; mandible	n/a; n/a	No	n/a
De Carvalho et al. [5]	18; M	0.5; mandible	+; +	No	1 year
Deboni et al. [6]	16; F	5; mandible	+; n/a	Yes	n/a
Gopalakrishnan et al. [7]	15; M	4.3; maxillary sinus	+; +	Yes	1 year
Leipzig et al. [8]	17; F	6; mandible	+; n/a	Yes	3 years
Maiorano et al. [9]	14; F	n/a; mandible	+; +	No	14 months
Mandal et al. [10]	17; F	6; hard palate	−; n/a	No	1 year
Mohanty et al. [11]	12; F	n/a; mandible	−; n/a	No	6 months
	13; F	n/a	−; n/a	No	6 months
Mopsik et al. [12]	13; F	n/a; maxilla	−; n/a	Yes	n/a
Rosa et al. [13]	17; F	n/a; mandible	+; +	No	7 years
Sharma et al. [14]	18; F	n/a; mandible	+; n/a	Yes	n/a
Ungari et al. [15]	9; M	0.8; maxilla	−; n/a	No	n/a

n/a: not available/applicable; “−” negative for Congo red stain; “+” positive for Congo red stain

## Case Report

**Fig. 1:**
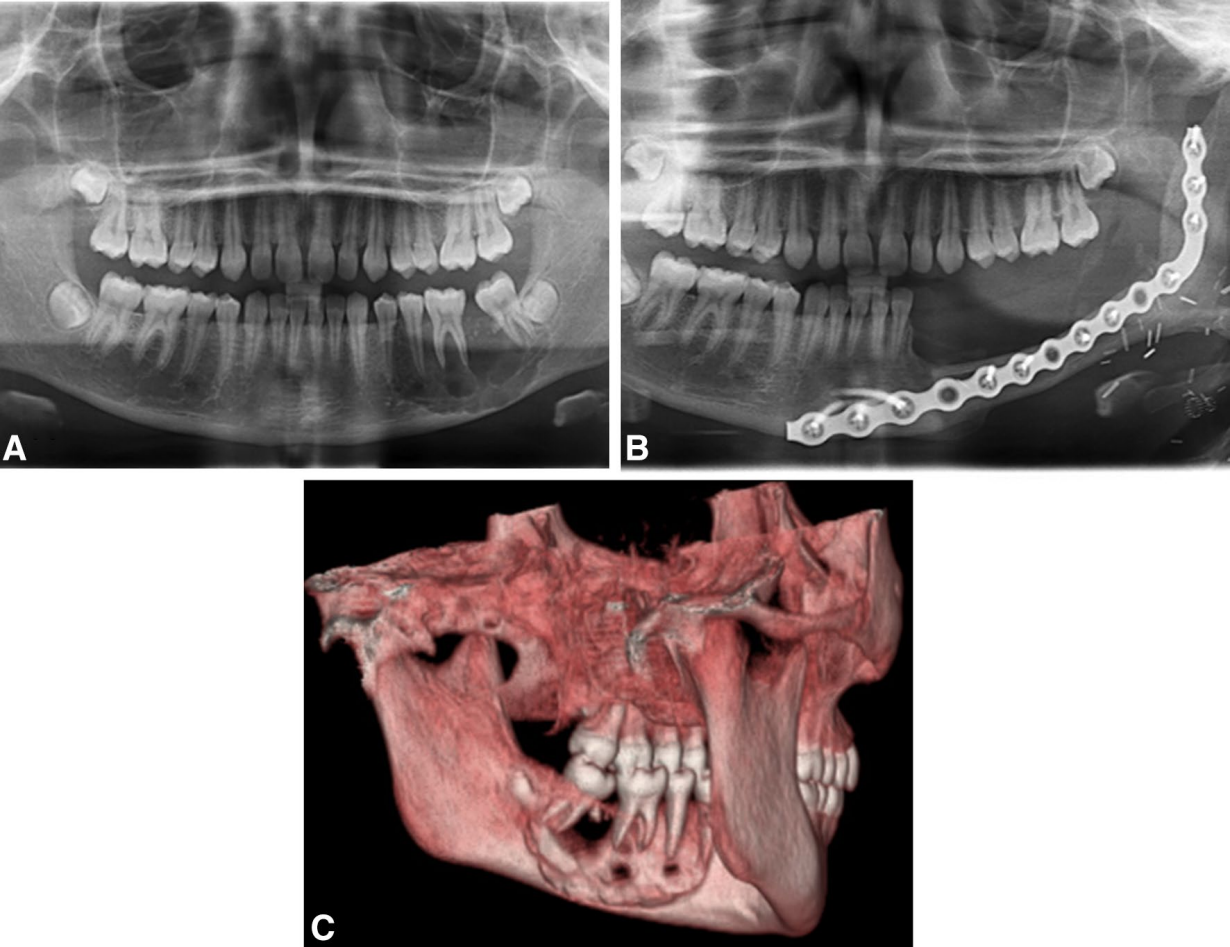
Pre-operative orthopantomogram: on the left lower jaw, a 3.8 × 1.5 cm expansile, radiolucent lesion with scattered punctate calcifications is seen in the premolar and molar teeth (**a**); post-operative orthopantomogram: a partial mandibulectomy with complete resection of tumor involved bone and teeth and fibula free flap reconstruction (**b**); pre-operative maxillofacial CT with 3-D reconstruction illustrated a 3.8 × 1.5 × 2 cm expansile radiolucent lesion in the left mandibular body and involvement of roots of teeth (**c**)

A 13-year-old female was incidentally found to have a large mandibular bone lesion during a routine dental visit. She was asymptomatic and had no complaints. There were no palpable lymph nodes on physical examination. The initial X-ray showed a lucent lesion with calcification. Orthopantomogram revealed an expansile, radiolucent lesion with scattered punctate calcifications in the mandibular body (Fig. 1a). Maxillofacial CT with 3-D reconstruction illustrated a 3.8 × 1.5 × 2 cm expansile radiolucent lesion in the left mandibular body and involvement of roots of teeth (Fig. 1c). A biopsy was performed at an outside hospital and initial impression was suggestive of a myxoid lesion. After further consultation, a diagnosis of CEOT was rendered. A left transcervical segmental mandibulectomy through an apron incision followed by mandibular reconstruction with left fibula free flap was performed at our institution. A segmental resection was performed in order to obtain a 1 cm clear margin at the inferior border of the mandible. Alternatively, a marginal resection in this case would potentially result in a close inferior margin and an exceedingly high risk for pathologic fracture. A limited ipsilateral neck dissection of levels I and II was performed purely for vessel access to facilitate the microvascular free flap and not for staging purposes or detection of possible metastasis given the benign nature of the lesion.

**Fig. 2:**
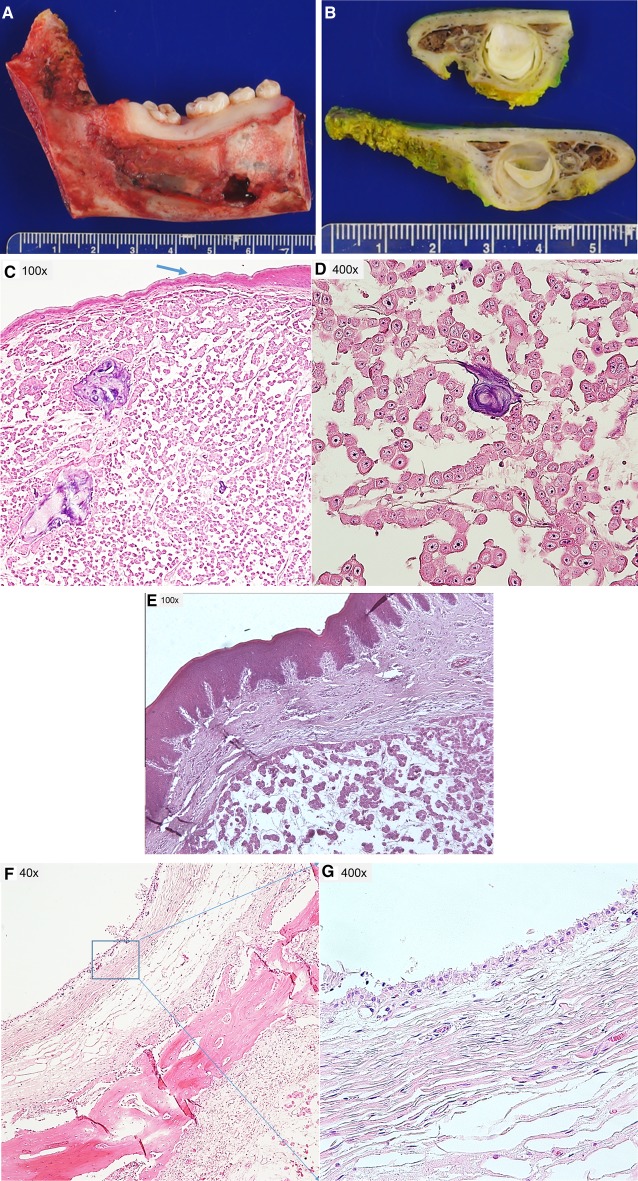
**a** Left segmental mandibulectomy specimen showing the cortical destruction by the tumor spanning teeth number 18–21. **b** Cross section of the developing molar tooth with associated tumor. **c** Low (× 100) and **d** high (× 400) power views showing the unique tumor morphology composed of monomorphic epithelioid tumor cells with distinct and prominent nucleoli and abundant eosinophilic cytoplasm with myxoid or mucinous stroma. The tumor is seen involving the mucosa (arrow) with islands of dystrophic calcification showing concentric lamellae. **e** A photomicrograph showing the pushing borders of the well circumscribed tumor and focal involvement of the gingival mucosa. **f** The odontogenic cyst associated with the tumor with the **g** columnar epithelial lining

Gross examination revealed a well-demarcated bone lesion (4.5 × 3.5 × 2.5 cm) occupying the mandibular body, extending and pushing into the cortical bone surface, associated with cortical thinning and destruction (Fig. 2a). The overlying gingival mucosa showed superficial erosion. The cut surface of tumor revealed mixed solid and cystic areas with granular and grey white tan myxoid textures. A cyst associated with a developing 3rd molar tooth was also found near the tumor (Fig. 2b).

Microscopic examination of the tumor revealed hypercellular areas composed of sheets of polyhedral epithelial cells alternating with hypocellular areas with cystic and degenerative spaces. The epithelial cells showed uniformly medium-sized nuclei with distinct and prominent nucleoli and abundant eosinophilic cytoplasm (Fig. 2c, d). Malignant features including nuclear pleomorphism, increased mitoses, necrosis and desmoplastic tissue reaction were not identified. The stroma was myxoid or mucinous with islands of calcification and frequent formation of concentric lamellae (Fig. 2c, d). Eosinophilic and amorphous deposits were present but negative for Congo red stain. The tumor was well-circumscribed with pushing borders and showed evidence of focal involvement of the gingival mucosa (Fig. 2e). An odontogenic cyst lined by ciliated columnar epithelium with stratified squamous epithelial component was seen near the developing third molar tooth consistent with a dentigerous cyts (Fig. 2f, g). Immunohistochemical stains for cytokeratins (AE1/AE3 and Cam 5.2) were used to confirm the epithelial origin of the tumor (picture not shown). The diagnosis of CEOT with extraosseous soft tissue involvement was made. All surgical margins were negative. The patient was followed up for 21 months after the procedure and showed no evidence of recurrence (Fig. 1b). Oral cavity exam showed excellent appearance of the flap.

## Discussion

CEOT is a rare tumor accounting for 1% of all odontogenic tumors [1, 25], usually seen in adults [1, 3, 11] with only 7% of the reported cases, including the present case, occurring in children. Tumors reported in the pediatric group tend to have variable presentation either as an asymptomatic/incidental lesion or a painful growth. Radiologic features of CEOT also vary depending on the stage of the tumor. At an early stage, the tumor may present as a radiolucent unilocular or multilocular (soap-bubble) lesion, whereas as the lesion progresses, radiopacities increase. The radiographic differential diagnoses include odontogenic myxoma, calcifying odontogenic cyst, complex odontoma, ameloblastic fibro-odontoma, fibro-osseous lesion and osteoblastoma. In the reported pediatric cases, the radiographic differential diagnoses included aneurysmal bone cyst, ameloblastoma, odontogenic keratocyst and dentigerous cyst [1, 5–15]. Although identifying the pathological entity based on radiological findings alone can be challenging, the overall tumor size, location and extension are important radiological clues for devising a plan for surgical intervention [19, 26, 27].

Histologically, CEOT is an encapsulated and non-invasive tumor with unique morphological features of discohesive clusters or floating tumor cells, like flower petals falling on the floor, without fibrous stromal reaction. Typically, like our case, the tumor cells are round to polygonal, with intermediate-sized centrally located nuclei and prominent nucleoli, distinct cell borders and abundant eosinophilic cytoplasm. Mitoses and nuclear pleomorphism are seldom seen [1, 19]. The matrix is myxoid or mucinous with islands of dystrophic calcifications, some showing concentric or psammomatous calcifications [2, 3, 25, 28].

In addition to the classic histologic appearance of the CEOT, the deposition of amyloid-like substance is another unique feature [1, 19]. There has been controversy over the origin of this homogenous material. El-Labban suggests the amyloid in CEOT is derived from degradation of lamina densa material, secreted by the tumor epithelial cells [29]. Page performed an ultra-structural study of CEOT which showed that the amyloid material is a protein product of the enamel organ completely different from those seen in endocrine-associated amyloid or systemic amyloid [30]. Amyloid-like material in CEOT shows green birefringence by Congo-red stain, which has been suggested as a useful stain for differentiating CEOT from other lesions [5]. However, in the present case, the eosinophilic homogenous material was negative for Congo red. Due to its affinity to mineral salts, the amyloid-like material can undergo calcification, causing the concentric appearance of lamellar bodies or Liesegang rings [1, 19, 31], which was seen also in our case.

One of the diagnostic challenges with our resection specimen was that the entire bone including the mucosal soft tissue was treated in a decalcifying solution, which may have affected the result of Congo-red staining. When working with bone tumors, it is essential to carefully dissect the tumor as much as possible before placing the entire bone specimen into decalcification solution. This is critically important, not only for better morphological preservation, but also for saving tissue for potential molecular studies for both diagnosis and management. Modified decalcification solution (with EDTA) is another option for softening the bone tissues. The difficulty is that this procedure takes longer than the current decalcification protocol, which prolongs the turnaround time by an additional 2–3 days.

Although CEOT is typically benign, its behavior varies depending on the histologic features and location. Necrosis, high proliferation index assessed by Ki-67, and nuclear pleomorphism are associated with a more aggressive behavior [23, 32]. Furthermore, involvement of the maxilla or the maxillary sinus is associated with rapid growth and invasion of the orbits and skull base [27]. Intraosseous involvement is another feature that is associated with higher chance of recurrence as compared to extraosseous tumor [27, 33]. In contrast, the presence of calcification and amyloid-like material indicates more differentiation and a lower likelihood of recurrence [34]. Malignant transformation and metastatic spread is extremely rare. To our knowledge, there have been 7 cases of either malignant CEOTs (n = 4) or with malignant transformation (n = 3) in patients between 40 and 83 years of age (Table 2). In addition to the conventional malignant features, other reported findings are vascular invasion, lymph node metastases or distant metastases (Table 2). Malignant transformation or aggressive features have not been reported in children (Table 1). However, due to rare occurrence of this tumor in children, the association of age with biological behavior of tumor cannot be clearly identified and long-term follow up is required. Among the cases reported in children (Table 2), one case of CEOT located in maxilla showed locally aggressive expansion to the lateral sinus wall, nasal cavity and orbital floor even though the tumor was incidentally found on routine dental examination. Provisional radiological diagnosis was dentigerous cyst, but the histologic diagnosis was a cystic variant of CEOT. There was no recurrence at 1-year follow-up. De Carvalho et al. reported a small CEOT (0.5 cm) in the mandible with benign behavior that did not recur 1 year after treatment [5]. Leipzig et al. described a 6 cm well-circumscribed mass in the mandible, which did not show any evidence of recurrence for 3 years [8]. Rosa et al. also presented an incidentally detected well-defined tumor causing the displacement of the roots of the neighboring teeth. The patient remained disease-free 7 years after the surgical excision [13]. Four other cases were followed up for up to 14 months and did not show recurrence [9–11]. In our case, the tumor showed locally invasive features including cortical bone destruction and involvement of the adjacent soft tissue; however neither histologic features of malignancy in the primary tumor nor recurrence over the 21 month after the surgical resection were seen.

**Table 2:** Reported cases of CEOT with invasive features or recurrence in adults

Authors	Age (year); gender	Size; location	Amyloid; Congo red	Impacted tooth	Invasive features; recurrence; metastasis; follow-up (if available)
Basu et al/ [35]	75; M	1 cm; mandible	+; +	No	Primary: pleomorphism, increased mitosis, lymph-node metastasis (6 years after resection)
Cheng et al. [36]	83; F	n/a; Mandible	+; +	No	Primary: clear cells, increased mitosis, pleomorphism, vascular invasion
Demian et al. [18]	45; F	4.5 cm; mandible	+; +	Yes	Primary: pleomorphism, increased mitosis, necrosis; recurrence 4 m after surgery and metastasis 10 month after resection
Goldenberg et al. [37]	40; M	n/a; mandible	−; n/a	No	Primary: malignant CEOT; no metastasis over 8 years
Kawano et al. [23]	54; M	n/a; mandible	+; +	No	Two recurrences: pleomorphism, frequent mitoses; vascular invasion; metastasis to lung after 3 years
Kumar et al. [24]	43; F	n/a; mandible	−; n/a	No	Recurrent: Clear cell odontogenic carcinoma; metastasis to spine 3 years after diagnosis
Veness et al. [22]	64; F	n/a; mandible	+; +	Yes	Malignant transformation 9 month after excision with vascular invasion, muscle infiltration, and lymph node metastasis

n/a: not available/applicable; “−” negative for Congo red stain; “+” positive for Congo red stain

One close differential diagnosis of CEOT is adenomatoid odontogenic tumor, frequently seen in young adults with a female predilection, mostly involving the maxilla. Radiological findings can be distinguishable from CEOT when there are features of radiopaque flecks (snowflake opacities). Histological features include a thick capsule with solid tumor nests and reticular pattern with duct-like structure and polygonal cells with pale to clear cytoplasm. Spindled and columnar cells can also be seen in adenomatoid odontogenic tumor. Ameloblastoma, a common odontogenic tumor more frequently seen in young adults, also shows clinical and radiological overlap with CEOT, albeit the different morphology. The classical ameloblastoma has follicular or plexiform patterns and is composed of islands of squamous epithelial cells with palisading basal cells and reverse epithelial polarity surrounded by dense stromal tissue.

The molecular biology of CEOT is not well understood. Mutation of *AMBN* (ameloblastin) gene has been found in CEOT as well as other tumors of odontogenic epithelium including ameloblastoma, adenomatoid odontogenic tumor and squamous odontogenic tumor, suggestive of a role of this mutation in tumorigenesis of the group of odontogenic tumors [38]. *PATCH1* gene mutation has also been found in both CEOT and keratocystic odontogenic tumors. However, the clinical significance of these mutations is unknown [39]. Demian et al. reported a case of CEOT with p53 gene mutation that presented with malignant transformation and distant metastasis [18], suggesting a potential tumor biomarker.

Depending on the location, size, and expansion of the tumor, method of treatment can range from enucleation or curettage to surgical resection. Minimizing the recurrence rate largely depends on the complete resection of the tumor [19, 25]. Recurrence rate of the tumor has been reported between 15 and 30% with higher rate in patients who underwent enucleation and curettage procedures [3, 25, 40]. Therefore, removal of the tumor with a 1 cm-negative margin is usually the preferred method of treatment regardless of tumor size. However, due to the more invasive nature of CEOTs in the maxilla and their proximity to vital structures, they require more aggressive surgical treatment. Tumors larger than 4 cm are treated by radical resection followed by reconstruction. Since there is high risk of recurrence if the tumor is incompletely resected, long-term follow up for at least 5 years is recommended [27, 33].

In summary, CEOT also known as Pindborg tumor is typically a benign yet locally aggressive tumor more commonly seen in middle-aged adults, but can be seen in children, albeit rarely. The pediatric CEOT follows a benign behavior track as in the majority of the adult cases. Amyloid-like matrix is a unique component that may be associated with tumor maturation and differentiation, and possibly lower risk of malignant transformation. Meticulous handling of the specimen with careful attempt to dissect the tumor involving the soft tissue helps preserve tumor morphology for accurate diagnosis and molecular studies. Malignant tumors or metastasis are extremely rare and have not yet been reported in children. However, malignant features need to be carefully examined and excluded on a well-sampled specimen. Whether soft tissue involvement plays any role in predicting prognosis is not clear. Recurrence rate is high with incomplete resection, which warrants resection with negative margins and a long-term follow up.

## Electronic supplementary material

Below is the link to the electronic supplementary material.

Supplementary material 1 (TIF 1434 KB)

Supplementary material 2 (TIF 2480 KB)

Supplementary material 3 (TIF 2455 KB)

Supplementary material 4 (TIF 2660 KB)

Supplementary material 5 (TIF 1888 KB)

Supplementary material 6 (TIF 1274 KB)
