# Supplementary figures and images for: Calcifying Epithelial Odontogenic (Pindborg) Tumor in a Child: A Case Report and Literature Review
Source: Head Neck Pathol. 2019 Feb 15;13(4):580–6. doi: 10.1007/s12105-019-01009-1 (PMC6853849; doi:10.1007/s12105-019-01009-1)

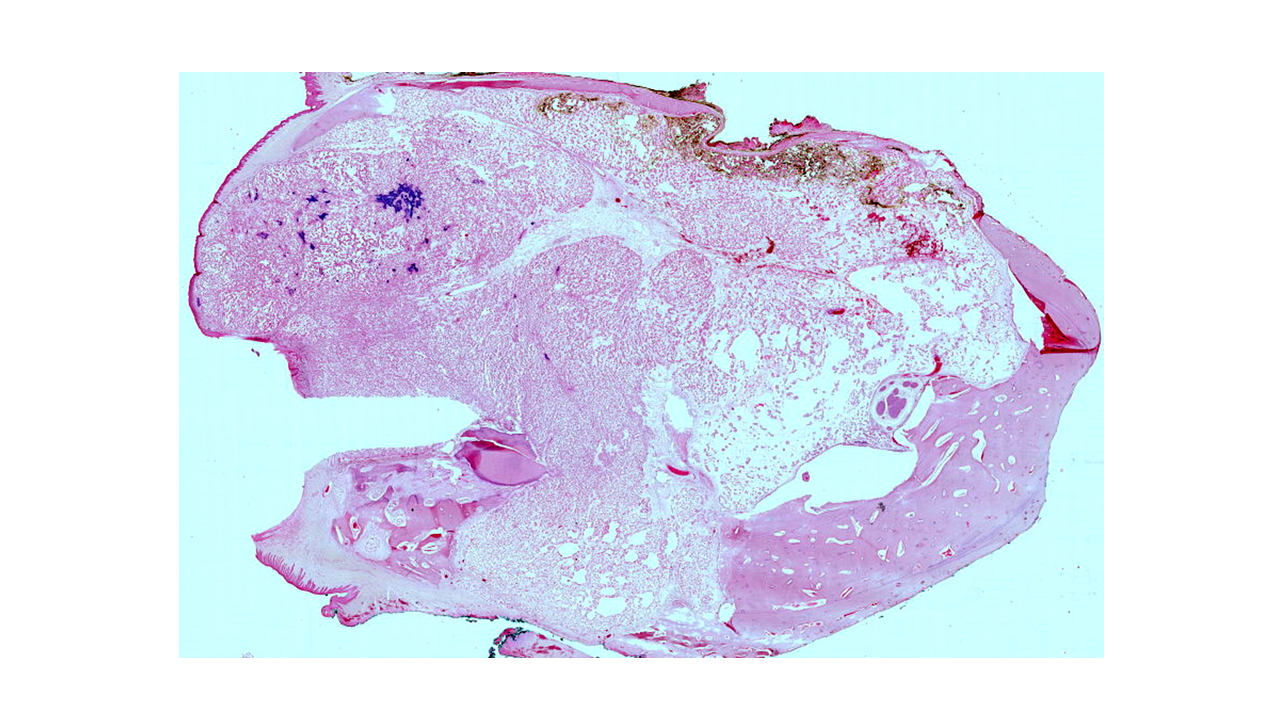

Supplement: Supplementary file 1 — Supplementary material 1 (TIF 1434 KB) [file 12105_2019_1009_MOESM1_ESM.tif]

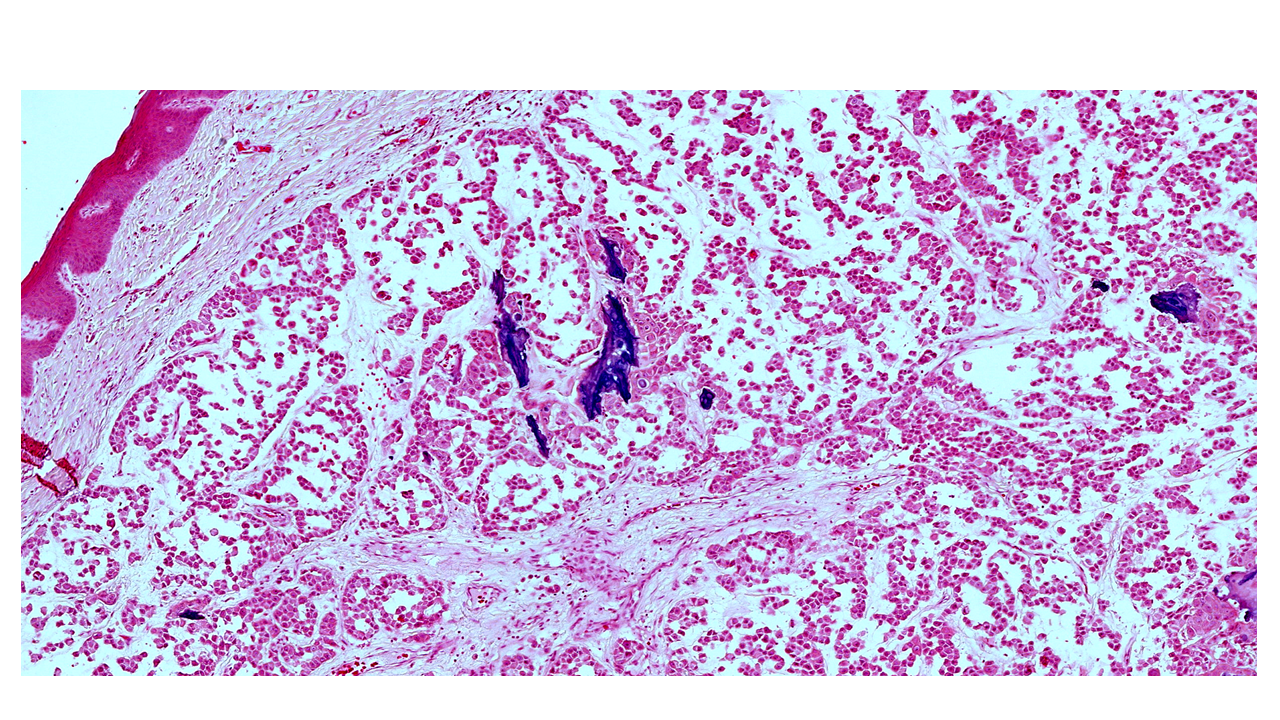

Supplement: Supplementary file 2 — Supplementary material 2 (TIF 2480 KB) [file 12105_2019_1009_MOESM2_ESM.tif]

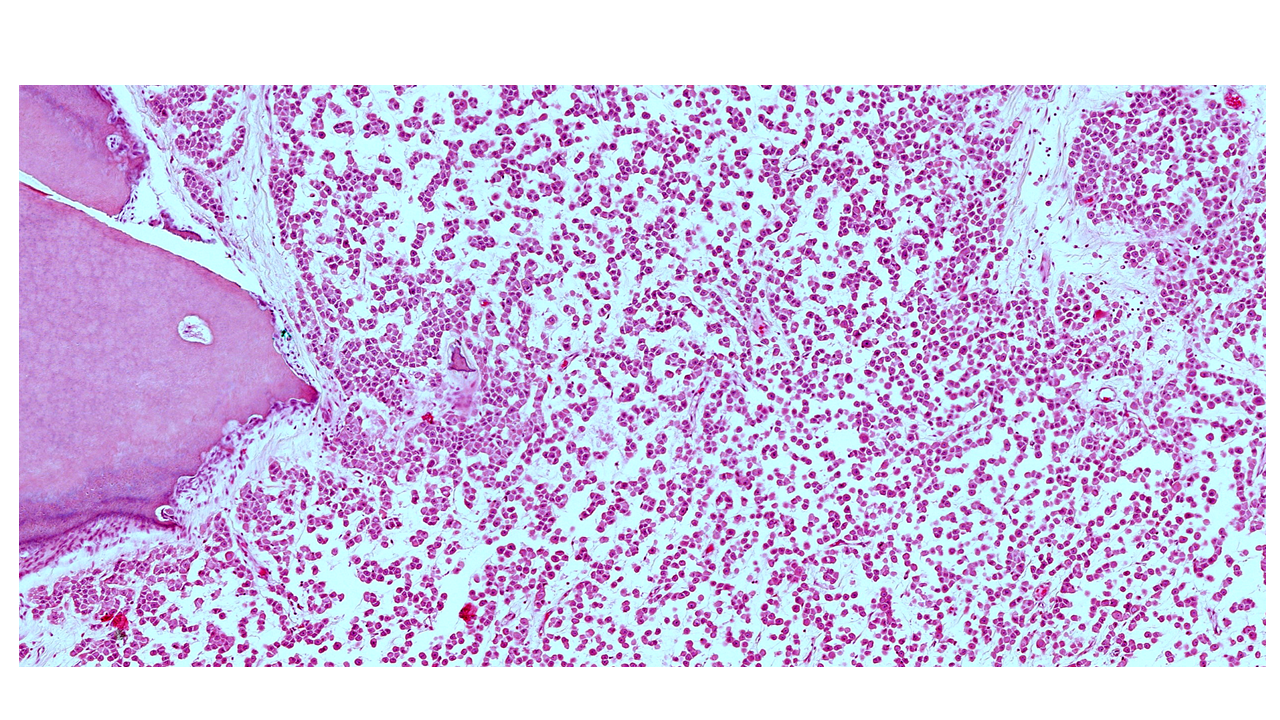

Supplement: Supplementary file 3 — Supplementary material 3 (TIF 2455 KB) [file 12105_2019_1009_MOESM3_ESM.tif]

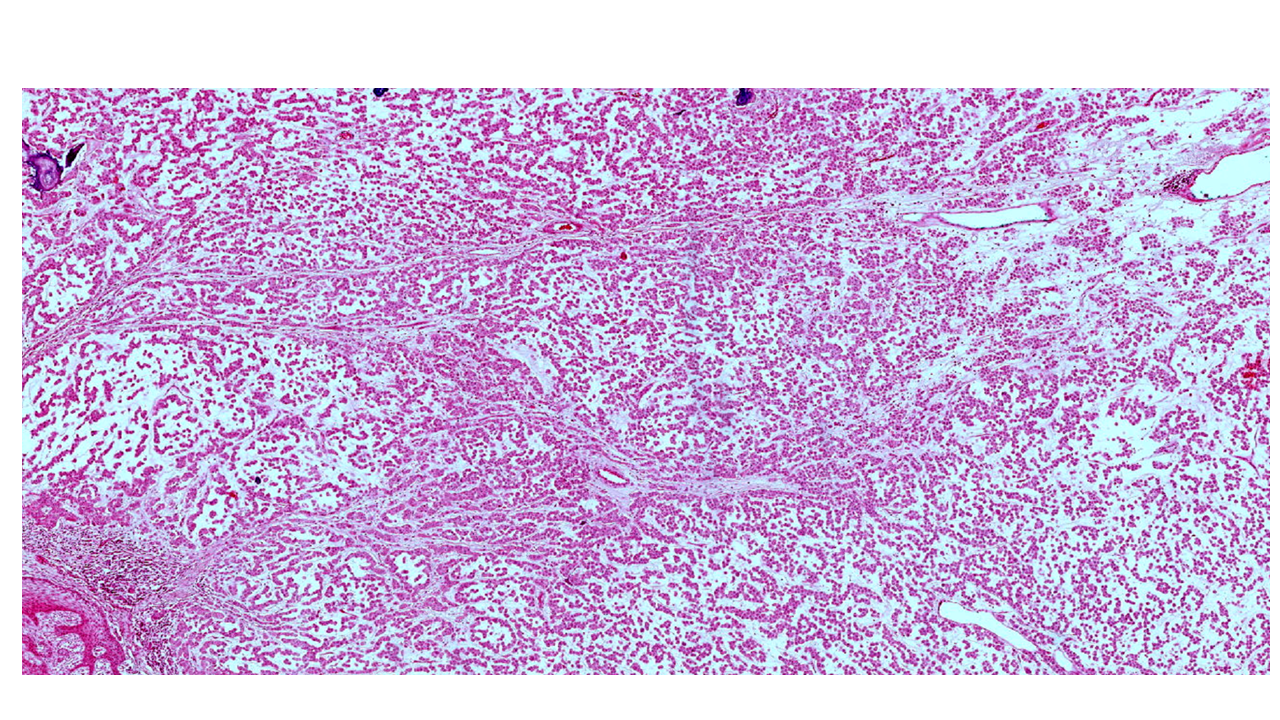

Supplement: Supplementary file 4 — Supplementary material 4 (TIF 2660 KB) [file 12105_2019_1009_MOESM4_ESM.tif]

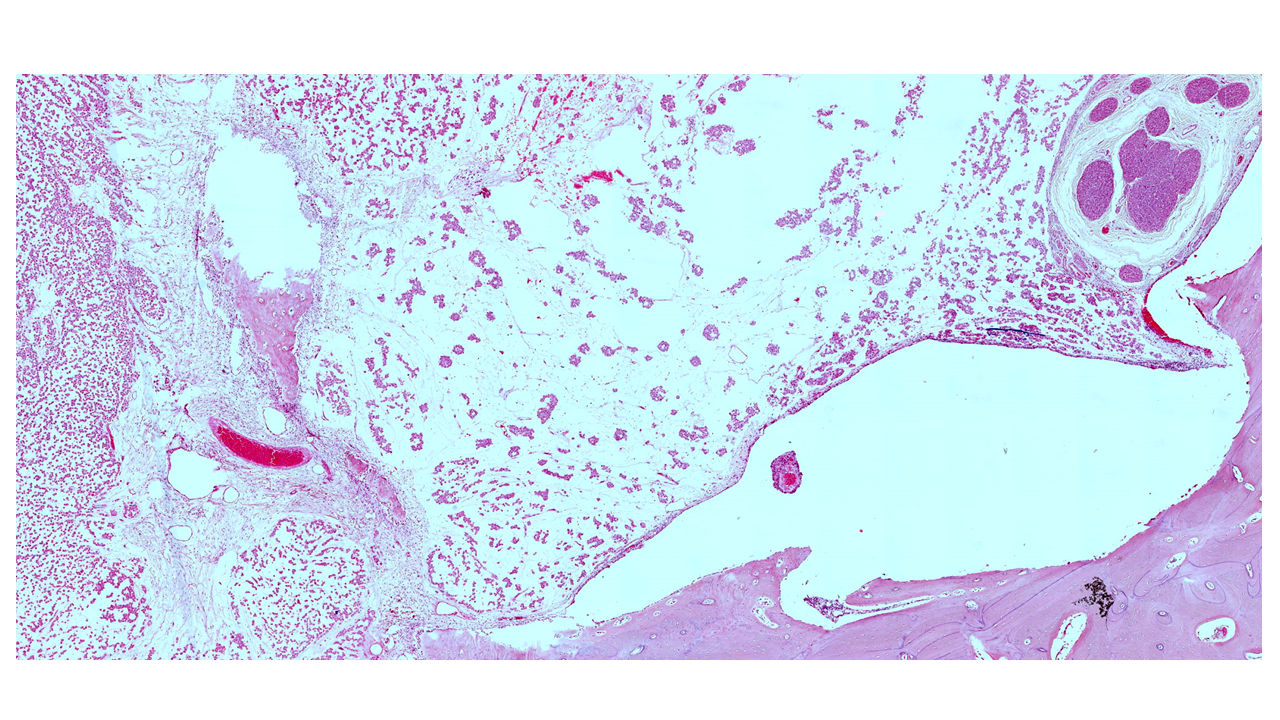

Supplement: Supplementary file 5 — Supplementary material 5 (TIF 1888 KB) [file 12105_2019_1009_MOESM5_ESM.tif]

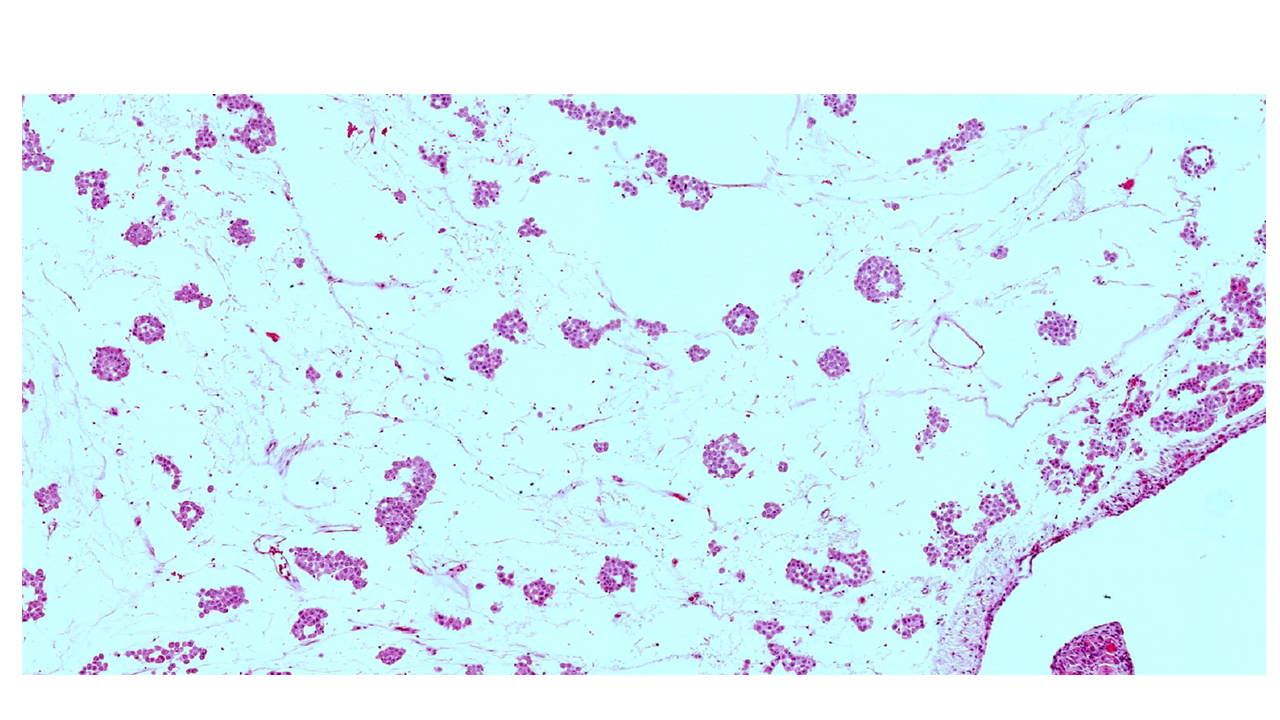

Supplement: Supplementary file 6 — Supplementary material 6 (TIF 1274 KB) [file 12105_2019_1009_MOESM6_ESM.tif]
